# Popularity of the metaverse: Embodied social presence theory perspective

**DOI:** 10.3389/fpsyg.2022.997751

**Published:** 2022-09-29

**Authors:** Guihua Zhang, Junwei Cao, Dong Liu, Jie Qi

**Affiliations:** ^1^Department of Business, Yeungnam University, Gyeongsan, South Korea; ^2^School of Business, Yangzhou University, Yangzhou, China; ^3^Department of Global Business, Yeungnam University, Gyeongsan, South Korea; ^4^Department of Sociology, Yeungnam University, Gyeongsan, South Korea

**Keywords:** metaverse, embodied social presence, embodied presence, embodied co-presence, imagination

## Abstract

With Facebook’s name changing to Meta, the metaverse concept has become popular again. There are many indications that the current fashionableness of the metaverse is not driven by technical factors, rather related to the public hype. To clarify the reasons for the increasing popularity of the concept, this study develops a model based on embodied social presence theory. We surveyed 292 metaverse users, and analyzed the obtained data using partial least squares structural equation modeling (PLS-SEM). The results show that the main technical factors influencing the metaverse popularity do not significantly predict users’ embodied presence and embodied co-presence, while users’ imagination positively predicts their embodied presence in the metaverse and positively influences users’ willingness to continuously participate through the multiple mediating effects of embodied presence and co-presence. The results of this study confirm, to some extent, that user imagination triggered by public opinion drives the popularity of the metaverse.

## Introduction

With the rapid development of virtual reality (VR) and augmented reality (AR) technologies in the past decade, people’s desire to live, learn and work in virtual worlds has grown (Al-Emran et al., 2020; Al Shamsi et al., 2022; Al-Sharafi et al., 2022). When Facebook CEO Mark Zuckerberg announced that Facebook would transform into Meta, the concept of a “metaverse” completely ignited the public’s interest in participating in this universal virtual world (Chen and Yao, 2021).

The metaverse is not a new concept, as it appeared in Neal Stephenson’s science fiction novel <<snow crash> > in 1992. The metaverse allows people to live and work in an immersive three-dimensional virtual world through virtual characters (Davis et al., 2009; Murray, 2020). Users can access the metaverse with the help of virtual and augmented reality devices. The metaverse promises greater overlap between real and virtual life in terms of economic innovation, social interaction, productivity enhancement, consumption, and entertainment (Bourlakis et al., 2009; Chen and Yao, 2021), thus enriching the development of both real and virtual societies.

The metaverse provides an immersive experience based on mixed reality (MR) technology, generates a mirror image of the real world based on digital twin technology (Boulos and Burden, 2007). and builds a virtual economic system, social system, and identity system based on blockchain technology (Park and Kim, 2022), which is a new type of Internet application and social form that integrates reality and reality by integrating multiple new technologies. Therefore, it is not difficult to find that the development and perfection of the metaverse requires the close integration of various technologies to provide technical support, while the extensive participation of users in the metaverse cannot be achieved without four technologies: communication technology, rendering technology, interaction technology and teamwork technology (Davis et al., 2009). Communication and interaction technologies underly the user experience in the metaverse, enabling users to create and exchange content, exchange digital assets in virtual worlds, and move between different virtual locations (Davis et al., 2009; Dionisio et al., 2013). The rendering technology affects users’ sense of immersion in the metaverse, as it determines the color fidelity, graphic fidelity, and 3D authenticity of users, buildings, terrain, trees, and other objects (Kumar et al., 2008; Dionisio et al., 2013; Hassouneh and Brengman, 2015). Teamwork technologies can enhance cross-regional collaboration experiences, such as creating a virtual world where real-life people in different geographic areas can collaborate and discuss, or even conduct experiments together to simulate real-world changes (Boulos and Burden, 2007). In addition to the aforementioned four technical points, the metaverse technology needs to achieve ubiquitous accessibility and scalability. Accessibility refers to whether the metaverse virtual space is accessible from all digital devices, and whether the user’s virtual identity remains constant across accesses (Dionisio et al., 2013). Scalability refers to whether the metaverse server provides sufficient operational capacity to allow a large number of users to use it simultaneously without compromising the efficiency of the system or user experience (Dionisio et al., 2013).

Research on the metaverse started before 2006, but the subsequent development was not satisfactory. In particular, attempts to integrate business into the metaverse failed (Hassouneh and Brengman, 2015). One of the main reasons for the failure was the poor virtual environment experience, especially when many avatars were clustered in the same area, frame drops and unresponsive controls severely affected the user experience and caused users to stop using it (Hassouneh and Brengman, 2015).

Therefore, is the current repopularization of the metaverse driven by current technological breakthroughs? The rapid development of deep learning technologies has significantly improved the accuracy of visual and speech recognition, providing a more immersive environment. The use of new end-to-end solutions reduces system processing time and complexity (Park and Kim, 2022). With the development of immersive interactive and blockchain technologies, the metaverse has played a greater role in fashion, gaming, education, and workplaces. In addition, It was previously based on a PC access, but now the ubiquitous accessibility problem has been solved by using mobile devices that can connect to the Internet quickly and at any time (Park and Kim, 2022). However, there are also studies that present opposing views. One study points out that the current metaverse technology is mainly implemented through VR technology, which gives the user a fragile sense of presence, and there is a huge contrast between the actual experience and the user’s imagination. Specifically, the current metaverse image quality is poor, the user’s movements are not accurately captured, and the user’s movements in the virtual world are limited, especially because the immersion created by the current technology for the user can be easily eliminated due to interference (Murray, 2020). Since the current impact of technological advances on user participation in the metaverse is not clear enough, it is necessary to further verify the mechanisms of how technology affects sustained user participation. From this, the first research question of this study is posed.

RQ1: How do technological factors influence users’ continuous participation and dominate this repopularization of the metaverse?

**Figure 1:**
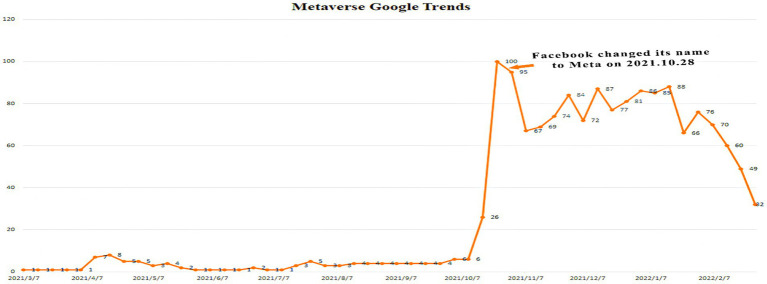
Metaverse Google trends.

Since the current impact of technological advances on user participation in the metaverse is controversial, do social factors then exist that influence the repopularization of the metaverse? Recent studies have investigated social factors that influence the repopularization of the metaverse. There are signs that the involvement of major technology companies, especially Facebook’s proposed metaverse transformation, has driven the public opinion (Lee, 2021; Cheng et al., 2022) and led users to participate in the metaverse b indulging in beautiful fantasies (Wang and Xiang, 2021). Before Facebook’s announcement of its name change, relevant information had leaked to the mass media. The starting point was to cope with the decline in consumer confidence and restore the company’s reputation by changing its name and using the innovation environment to reshape the brand (Çelikkol, 2022). According to Google Trends data (Figure 1), this global explosion of interest in the metaverse coincided with the timing of Facebook’s name change, which might indicate that the public opinion is driving user engagement.

In summary, the dominant factors of the current metaverse repopularization are not sufficiently clear. First, there are studies with opposing views on the impact of current technology on user engagement (Murray, 2020; Park and Kim, 2022). Second, there are indications that opinion orientation may influence users’ engagement psychology, but there is a lack of empirical studies clarifying how these factors contribute to the development of the metaverse. Therefore, the questions posed in this study are as follows:

RQ2: Is the popularity of the metaverse driven by technological or psychological factors?

## Theoretical background

### Embodied social presence theory

**Figure 2:**
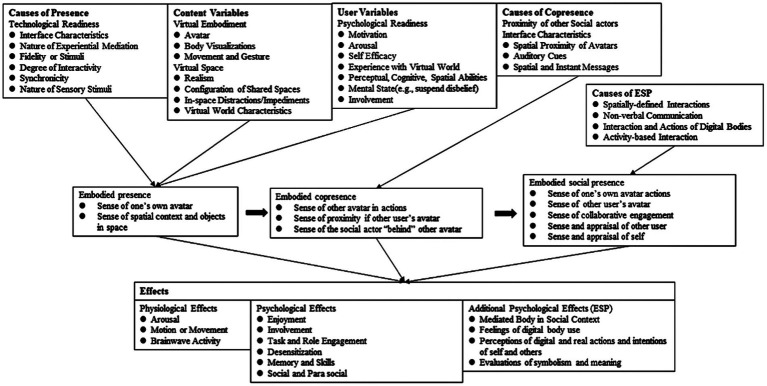
Embodied social presence theory.

Mennecke et al. (2010) proposed the embodied social presence (ESP) theory (e.g., Figure 2), which focuses on avatars as mediators of social interactions in virtual worlds. In the context of embodiment, the occurrence of specific acts of communication and interaction creates a sense of presence that is derivative of human cognition and similar to real interactions in the real world (Wang et al., 2016). The core of the theory is that in a virtual world, users must first feel the existence of their own avatars, then through interaction with other avatars, they feel a common existence with others and generate a sense of social presence in the virtual world (Mennecke et al., 2010). However, to achieve ESP, a person must first achieve an adequate level of perceived presence and co-presence (Mennecke et al., 2011).

Embodied presence comprises technical preparation, content variables, and user variables (Mennecke et al., 2011). Technical readiness is focused on the interface characteristics, experience, reality of the virtual world, quality of interaction, and sensory stimulation. Technical quality allows the user to be mentally and emotionally immersed in another world, improving the user’s sense of presence in his or her own avatar in the virtual world, while also enhancing the sense of space and objects in the virtual world. Content variables are the types of specific content that are generated by the technology, such as the appearance of the user avatar, form when moving, and obstacles in the virtual world. The presence of these content variables allows users to feel the similarity between the virtual and real worlds, deepening their sense of immersion. User variables are also important factors for increasing the user’s perception of the presence of an avatar and are related to the user’s experience, mental state, perceived ability, and self-efficacy (Mennecke et al., 2011).

When a user who generates a sense of embodied presence feels his or her presence in the virtual world by receiving messages (either verbal or nonverbal) from other avatars, the user enters a state of embodied co-presence. After generating a sense of embodied presence and co-presence, he or she feel a social presence in the virtual world through interaction and co-participation, thus generating a sense of themselves and others. In addition, under the influence of embodied presence, users develop physiological and psychological effects such as collaborative participation, establishing quasi-social relationships, and enjoying virtual social life (Mennecke et al., 2011).

The ESP theory provides a framework within which user interactions in virtual worlds can be studied. This theory analyzes behavior in virtual worlds from a human psychological perspective, clarifying the different stages of social presence in virtual worlds. Consequently, it has been widely applied in the study of virtual worlds (Wang et al., 2016, 2018). As the metaverse is a three-dimensional virtual world in which people can interact as avatars without physical constraints (Davis et al., 2009), this theoretical framework can be applied to the study of user behavior in the metaverse.

### Metaverse research framework

**Figure 3:**
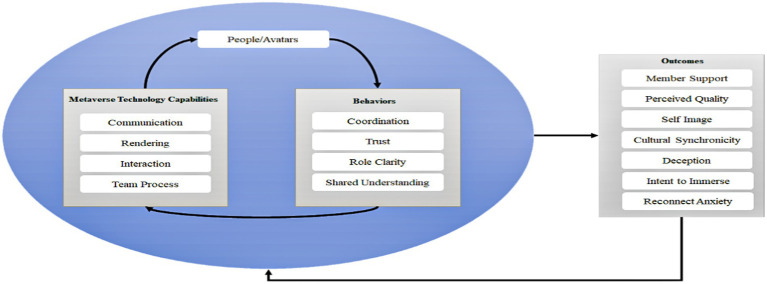
A diagram for the Davis et al. (2009) framework for the study of the metaverse.

Davis et al. (2009) proposed a conceptual framework as a basis for studying the metaverse after incorporating unique technological capabilities and behaviors in a metaverse environment. They studied teamwork behavior in a virtual environment (as shown in Figure 3). This framework has five parts: the metaverse itself, the person and the avatars, the technological capabilities of the metaverse, the behavior, and the outcomes. The circular relationship between the metaverse and the outcome illustrates that the metaverse is influenced by technological capabilities and continuous social interactions. This circular relationship indicates that these structures interact with each other rather than reflecting a one-way causal relationship (Davis et al., 2009).

In other words, the technological capabilities of the metaverse determines the quality of interaction between a person and his avatar, which leads to the mental or behavioral aspects of the avatar in the metaverse, such as trust, sharing, and understanding. The psychological state and behavior of avatars will, in turn, facilitate communication and interaction, continuously improving the virtual environment of the metaverse and eventually producing various results. The framework suggests that metaverse technologies must have the ability to communicate, render, interact, and provide teamwork tools that can influence the representation of people and their incarnations in the metaverse, such as presence and immersion (Davis et al., 2009). This influence mechanism is consistent with the influence of technology readiness on users’ embodied presence as proposed in the ESP theory. However, as this study focuses on individual participation rather than teamwork in the metaverse, we only consider the communication, rendering, and interaction capabilities in light of the ESP theory and examine their effects on embodied presence and co-presence.

## Research model and hypothesis development

### Research model

**Figure 4:**
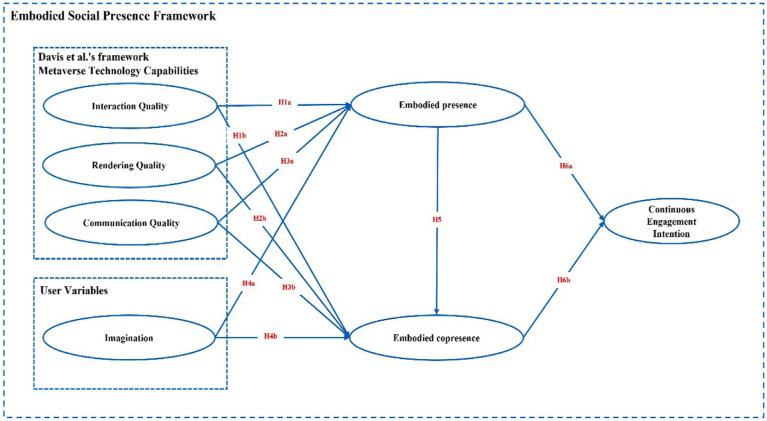
Research model.

The Davis et al. (2009) research framework does not consider the psychological component of the user compared to the ESP theory, which fills this gap by suggesting that the psychological component of the user variable has a positive impact on the user’s embodied presence (Mennecke et al., 2011). Therefore, according to the actual situation in this study, the user variable was set as a psychological factor (i.e., imagination of the metaverse) to examine its effect on the embodied presence and co-presence of the user. Davis et al. (2009) suggested that the psychological sensation of user incarnation affects the user’s behavior or psychological state, such as trust, shared understanding, and clarity of roles; while the ESP theory suggests that the sense of presence or co-presence also affects the user’s physiology and psychology, such as participation, enjoyment, and establishment of prosocial relationships. In addition, the ESP theory suggests that users’ embodied presence has a positive effect on embodied copresence (Davis et al., 2009). Thus, the current study tests such a relationship in the context of the metaverse and ultimately proposes a research model, as shown in Figure 4.

### Hypothesis development

Interaction is often associated with the design of computer-based products and systems. It is an issue that describes the usability of a computer product and is actively related to the way people interact with the system. The quality of interaction determines when and how users connect with a computer (Hallnäs, 2011), and is considered a key component of service quality (Ekinci and Dawes, 2009). Interactivity in the metaverse includes the ability of users to participate, modify, and create virtual environment content in real time (Davis et al., 2009). Interaction is composed of the interactivity, mobility, and immediacy of the metaverse system, which positively affects the user’s sense of remote presence (Davis et al., 2009). Social presence theory focuses on mediated social interaction (Weidlich and Bastiaens, 2017), with some studies pointing out that there are three interactive dimensions of social presence: consumer-consumer, consumer-merchant, and consumer-goods interactions (Zhang et al., 2021). Additionally, these studies showed that interactivity, social environment, and online communication have a positive impact on the sense of presence (Park and Kim, 2022).

Interaction and co-presence are also related. Co-presence can be measured by user engagement with the environment (Wu et al., 2021). When interacting with others, co-presence refers to how one perceives his or her own participation and the participation of others in the interaction (Tang and Bradshaw, 2020). Research has shown that interaction needs to be emphasized when generating a sense of co-presence (Taani et al., 2020).

In a metaverse environment, the better the quality of interaction provided by the metaverse technology, the more realistic the user will be able to interact with the avatars instantly and with the metaverse environment; and the higher the quality of interaction, the more the user will be able to interact with other people’s avatars in multiple ways. Therefore, this study proposes the following hypotheses:

*H1a*: The quality of interaction provided by the metaverse technology has a positive impact on the user’s embodied presence.

*H1b*: The quality of the interaction provided by the metaverse technology has a positive impact on the user’s’ sense of embodied co-presence.

The rendering technology involves creating lifelike images on a screen; thus, it requires personalization and vividness (Davis et al., 2009). Personalization allows users to better customize their avatars and distinguish others’ avatars based on their appearance. Vividness represents the richness of the media environment. The rendering technology must provide a higher level of vividness, by visually presenting the real or virtual environment to the user clearly. This suggests that the better the quality of rendering, the richer the user’s sensory experience in the metaverse, and the more realistic the interaction with others. Empirical studies show that presence increases with vividness; the closer the virtual and real environments, the higher the user’s sense of presence (Zimmons and Panter, 2003; Nicovich et al., 2006; Van Kerrebroeck et al., 2017; Kim and Ko, 2019). The same is true for the sense of co-presence. Some studies have pointed out that the physical rules of the real environment are correctly rendered in the virtual environment through rendering techniques, which increase the sense of co-presence for the users (Kim et al., 2020). Therefore, we postulate the following hypotheses:

*H2a*: The rendering quality of metaverse technologies has a positive impact on the user’s embodied presence.

*H2b*: The rendering quality of metaverse technologies has a positive impact on the user’s sense of embodied co-presence.

Communication is the basis for interaction and collaboration, and its quality can be enhanced by improving openness, efficiency, and effectiveness (Lowry et al., 2009). Davis et al. (2009) noted that the technological capabilities associated with communication in the metaverse include feedback, multiple cues and channels, linguistic diversity, and communication support technologies. Social presence and communication theories are based on interpersonal communication (Yue et al., 2019). Communication promotes awareness of others and builds interpersonal relationships to create a sense of social presence for the user (Gu et al., 2019). Users in a community can consistently engage in trustworthy, polite, and open communication that helps them develop a sense of social presence (Ekasari et al., 2020). In social communication, a sense of common existence is formed when there is spiritual connection with others (Kadylak et al., 2018). Similarly, as a virtual community, the metaverse may also apply to such relationships. Therefore, we put forward the following hypotheses:

*H3a*: The quality of communication of metaverse technologies has a positive impact on the users’ embodied presence.

*H3b*: The quality of communication of metaverse technologies has a positive impact on the users’ sense of embodied co-presence.

Mass media are adept at compensating for the lack of reality by channeling users’ fantasies (Crippen et al., 2010). In particular, digital media emphasize the importance of certain issues and the impact of imagination on human action (Sulistyanto et al., 2019). Additionally, imagination will be popular in communities if it can provide some level of evidence or logic to solve problems (Underation, 2012). The repopularization of the metaverse began with the strategic layout of large companies, then it was widely disseminated through the mass media, driving the public to fantasize about the metaverse and participate in many metaverse communities. Imagination and presence have been linked (Pellegrini, 2001). One study suggested that learners who experience imagination through multimedia presentations might have a stronger sense of presence because they are able to see, hear, and even move their characters in a fantasy environment (Jessen and Renee., 2008). When the public learns about and participates in metaverse communities, driven by the media hype, they may have a strong sense of presence due to fantasy. Another study noted that users in a metaverse environment could also customize each other’s avatars based on their imagination, thus increasing the mutual perception of each participant and consequently the sense of co-presence (Wurtz et al., 2013). Therefore, we propose the following hypotheses:

*H4a*: In the metaverse, the user’s imagination has a positive effect on his or her embodied presence.

*H4b*: In the metaverse, the user’s imagination has a positive effect on his or her sense of embodied presence.

The ESP theory states that users experience the metaverse using avatars to participate in shared activities and gain a sense of embodied presence (Mennecke et al., 2010). In other words, avatars provide users with a sense of embodied presence by forming bonds through avatar interactions, building relationships with others, and ultimately experiencing a sense of corporate presence (Mennecke et al., 2011). The emergence of a sense of embodied presence and co-presence affects the physiological and psychological aspects of the individual, such as role involvement, enjoyment, and the establishment of prosocial relationships. Therefore, we put forward the following hypotheses:

*H5*: In the metaverse, the user’s embodied presence has a positive effect on his or her embodied co-presence.

*H6a*: In the metaverse, the user’s embodied presence has a positive impact on his or her continuous engagement intention.

*H6b*: In the metaverse, the embodied co-presence of the user has a positive impact on his or her continuous engagement intention.

## Questionnaire survey and empirical analyses methods

### Questionnaire design and survey

We designed a questionnaire suitable for this study based on the scales proposed by existing studies. The questionnaire used a 5-point Likert scale. Experts in the field revised it. The final version of the questionnaire is shown in Appendix A.

Data from the Baidu Index^1^ indicated that in mid-January 2022, residents of Shenzhen, Guangdong Province, China were most concerned about the metaverse. Therefore, we conducted a questionnaire survey between 8 February and 28 February 2022, among the residents of Shenzhen, Guangdong Province, China. The questionnaire survey followed the recommendations of the university’s scientific ethics review committee and was designed in an ethics-free manner. Anonymous surveys were used, and participants were informed of the purpose of the survey. Only necessary data were collected and kept strictly confidential, and certain incentives were given after the survey.

### Structural equation model

There are two types of structural equation models: covariance-based structural equation models (CB-SEM) and variance-based structural equation models (VB-SEM). In this study, we used variance-based partial least squares structural equation modeling (PLS-SEM) with the corresponding software package SmartPls 3.0 for data analysis. The main reasons for this were as follows: (1) PLS-SEM is more suitable for measuring complex models than CB-SEM, especially those with more than six variables (Hair et al., 2017), and this study used seven variables; (2) PLS-SEM can better calculate non-normally distributed data, compared to CB-SEM (Hair et al., 2017). A multivariate normality analysis was performed on the data using a web-based calculator^2^ (Korkmaz et al., 2014). The results show Mardia’s multivariate skewness (β = 44.078, *p* < 0.05) and multivariate kurtosis (β = 488.627,n.s.), indicating multivariate non-normality (Sharma et al., 2021); (3) PLS-SEM is more suitable for measuring small samples (Hair et al., 2017). In conclusion, PLS-SEM was more suitable for the data analysis of this study.

## Results

### Demographics and bias results

The questionnaire was sent to the participants through Amazon Simple Notification Service (SNS), and 456 answers were collected. After removing invalid answers from respondents who did not know the metaverse, did not use metaverse applications, and those who repeated their answers, 292 valid answers were obtained (64%). Among them, 156 (53.4%) were male and 136 (46.6%) were female; 137 (46.9%) aged between 20 and 29 years, 78 (26.7%) aged between 30 and 39 years; and 98 (33.6%) had a specialist degree, 94 (32.2%) had a bachelor’s degree. The majority of respondents earned RMB 0–1999, with 88 (30.1%) having an income ranging between RMB 4,000 and 5,999, followed by 76 (26.1%). In terms of metaverse applications, 122 people used “Creator City” (41.8%), 61 people used “Zepeto” (20.9%), 52 people used “Roblox “(17.8%), and 57 people used other applications (19.5%).

To avoid nonresponse bias, we performed a paired t-test on the demographic data of the first and last 20 people who answered the questionnaire. The results showed no significant difference; therefore, nonresponse was not a serious problem in this study.

In this study, two methods were used to measure common method bias. (1) Harman’s single-factor analysis (Podsakoff et al., 2003), was conducted. The results showed that the percentage of variables extracted was 19.941% (<50%). (2) The common method bias in PLS-SEM was measured according to the FLL-VIF (Kock, 2015; Sharma et al., 2021). All VIF values were below 3.3. The results of both testing methods suggest that the common method bias was not a serious problem in this study.

### Measurement model result

First, we assessed the reliability validity of the model. As shown in Table 1, the composite reliability of the variables was >0.7, and Cronbach’s alpha was >0.7, confirming the internal consistency of the data in this study (Hair et al., 2017). The AVE of the variables was >5 and that of outloadings was >0.7, confirming the convergent validity of the data in this study (Hair et al., 2017). As shown in Table 2, the discriminant validity of this study was measured using Fornell and Larcker’s Test and the Heterotrait-Monotrait ratio (HTMT) Test. The HTMT values between the variables were below the 0.85 threshold, and the square root of each variable AVE was also greater than the correlation between the variables (Hair et al., 2017), confirming the discriminant validity of this study. The results of the above analysis showed that the study had good overall reliability, convergent validity, and discriminant validity.

**Table 1:** Reliability and validity coefficients for constructs.

Latent variable	Item	Loading	Mean (SD)	Cronbach’s a	CR	AVE	R^2^
INQ	INQ1	0.781	3.013 (1.082)	0.827	0.859	0.671	
	INQ2	0.740					
	INQ3	0.925					
REQ	REQ1	0.823	3.014 (1.041)	0.870	0.908	0.767	
	REQ2	0.875					
	REQ3	0.927					
COQ	COQ1	0.912	3.139 (1.119)	0.846	0.903	0.757	
	COQ2	0.890					
	COQ3	0.803					
IMA	IMA1	0.754	3.050 (1.030)	0.758	0.862	0.677	
	IMA2	0.885					
	IMA3	0.823					
EPO	EPO1	0.863	2.763 (0.716)	0.745	0.854	0.662	0.264
	EPO2	0.764					
	EPO3	0.811					
ECP	ECP1	0.777	2.647 (0.777)	0.767	0.866	0.685	0.175
	ECP2	0.909					
	ECP3	0.790					
COE	COE1	0.902	2.389 (0.682)	0.791	0.868	0.689	0.228
	COE2	0.764					
	COE2	0.817					

INQ, interaction quality; REQ. rendering quality; COQ, communication quality; IMA, imagination; EPO, embodied presence; ECP, embodied co-presence; COE, continuous engagement intention.

**Table 2:** Discriminant validity.

**Fornell-Larcker criterion**							
	INQ	REQ	COQ	IMA	EPO	ECP	COE
INQ	0.819						
REQ	−0.117	0.876					
COQ	0.042	0.147	0.87				
IMA	0.028	−0.04	0.027	0.822			
EPO	0.03	−0.045	0.077	0.515	0.814		
ECP	0.073	0.008	0.04	0.258	0.388	0.828	
COE	0.081	0.031	0.076	0.194	0.279	0.467	0.830
**Heterotrait-monotrait ratio**							
	INQ	REQ	COQ	IMA	EPO	ECP	COE
INQ							
REQ	0.105						
COQ	0.096	0.167					
IMA	0.055	0.045	0.123				
EPO	0.056	0.064	0.089	0.673			
ECP	0.078	0.039	0.067	0.338	0.522		
COE	0.068	0.084	0.104	0.212	0.343	0.54	

INQ, interaction quality; REQ, rendering quality; COQ, communication quality; IMA, imagination; EPO, embodied presence; ECP, embodied co-presence; COE, continuous engagement intention.

### Structural model result

**Figure 5:**
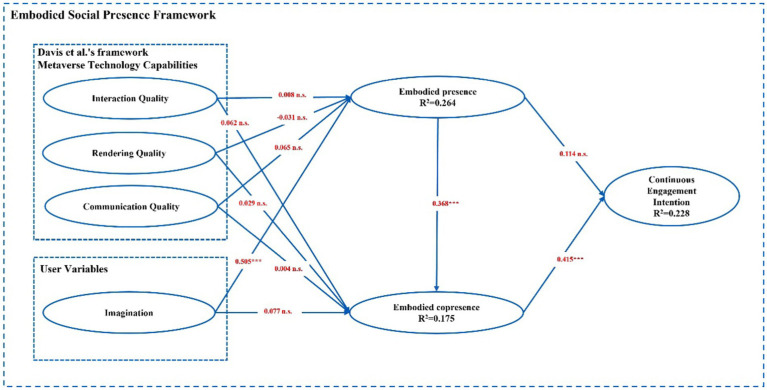
Test results of the structural model test. ^*^p < 0.05, ^**^p < 0.01, ^***^p < 0.001.

Then, we measured the covariance problem. The VIFs of the variables were all below 5; thus, covariance was not a major issue in this study. After ensuring that the reliability, validity, and covariance of the model did not pose a problem, this study analyzed the structural model to test our hypotheses. The results of testing the path coefficients and significance cases of the structural model are presented in Table 3. We found that interaction quality, rendering quality, and communication quality have no restricted positive effects on embodied presence and embodied co-presence, thus refuting H1a, H1b, H2a, H2b, H3a, and H3b. The results showed a significant positive effect of imagination on embodied presence (β = 0.505, *p* < 0.001), thus confirming H4a. However, we found no significant positive effect of imagination on embodied co-presence, thus refuting H4b. Embodied presence had a significant positive effect on embodied copresence (β = 0.368, *p* < 0.001), thus confirming H5. Embodied presence had no significant positive effect on continuous engagement intention, thus refuting H6a. Embodied co-presence had a significant positive effect on continuous engagement intention (β = 0.415, *p* < 0.001); thus confirming H6b (Figure 5).

**Table 3:** Assessment of the structural model.

Hypothesis	β	STDEV	*T* Statistics	*p*-Values	Result
H1a: INQ → EPO	0.008	0.069	0.119	0.905	Reject
H1b: INQ → ECP	0.062	0.083	0.751	0.453	Reject
H2a: REQ → EPO	−0.031	0.054	0.57	0.569	Reject
H2b: REQ → ECP	0.029	0.07	0.421	0.674	Reject
H3a: COQ → EPO	0.065	0.057	1.147	0.251	Reject
H3b: COQ → ECP	0.004	0.065	0.06	0.953	Reject
H4a: IMA → EPO	0.505	0.048	10.596	0.000	Support
H4b: IMA → ECP	0.077	0.07	1.108	0.268	Reject
H5: EPO → ECP	0.368	0.086	4.272	0.000	Support
H6a: EPO → COE	0.114	0.067	1.711	0.087	Reject
H6b: ECP → COE	0.415	0.063	6.61	0.000	Support
Gender → COE	−0.001	0.053	0.013	0.989	–
Age → COE	−0.01	0.051	0.204	0.838	–
Income → COE	0.041	0.059	0.689	0.491	–
Edu → COE	0.013	0.056	0.234	0.815	–
Platform-G-COE	0.027	0.053	0.497	0.619	–

INQ, interaction quality; REQ, rendering quality; COQ, communication quality; IMA, imagination; EPO, embodied presence; ECP, embodied co-presence; COE, continuous engagement intention.

We also tested the goodness of fit (GOF) of the model, although according to the properties of PLS-SEM, PLS-SEM itself does not need and does not have a reliable way to accurately measure the fit of the model. However, earlier studies noted that a global criterion of goodness-of-fit (GoF) can be proposed as the geometric mean of the average communality and average R^2^. That is, the square root of the mean of R^2^ and the mean of communality can be used to measure the fit of the PLS-SEM model (Tenenhaus et al., 2005). A goodness of fit greater than 0.36 can be classified as “upper;” 0.25–0.36 as “middle,” and 0.1–0.25 as “lower” (Cao et al., 2021). The GOF value for this study was 0.270. Later studies pointed out that the standardized root mean square residual (SRMR) values could be used to check the fit of the PLS-SEM model (Benitez et al., 2020), with this method being widely used (Sharma et al., 2021). The SRMR value of our model was 0.069, which meets the requirement of less than the threshold value of 0.08 (Benitez et al., 2020). Combining these two indicators, it can be concluded that the fit of our model is adequate.

### Mediation effect

Based on the measurement results of the structural model, to determine how the user variables in this study affect sustained engagement, we analyzed the mediating role in the model using SmartPls. The mediating effects are shown in Table 4. Embodied presence had a significant mediating effect on the user’s fantasy and embodied co-presence (β = 0.186, *p* < 0.001). There was a significant mediating effect of embodied co-presence on the relationship between embodied presence and continuous engagement intention (β = 0.077, *p* < 0.05). We also found a multiple mediator in the model, that is, imagination had a significant effect on continuous engagement intention after the mediation of embodied presence in embodied co-presence.

**Table 4:** Assessment of mediation effect.

Path	β	STDEV	*T* Statistics	*p*-Values
IMA → EPO → ECP	0.186	0.046	4.057	0.000
EPO → ECP → COE	0.152	0.047	3.258	0.001
IMA → EPO → ECP → COE	0.077	0.025	3.034	0.002

IMA, imagination; EPO, embodied presence; ECP, embodied co-presence; COE, continuous engagement intention.

## Discussion and implications

### Research findings

The primary purpose of this study is to clarify whether the current repopularization of the metaverse is driven by technical or user psychological factors. The findings confirm that technical factors (i.e., interaction, rendering, and communication) do not significantly predict the users’ embodied presence and co-presence in the metaverse, nor do they predict the users’ continuous engagement intentions through the mediating effect of embodied presence and co-presence, that means that the current re-popularization of the metaverse is not dominated by technology factors. This may be due to the fact that the metaverse is still in the stage of technological development and perfection, and the current technical factors of the various metaverse apps do not predict the continuous engagement of users with the metaverse. The current technology in the metaverse is not yet able to maximize user satisfaction, such as the low quality of metaverse images and interaction makes it difficult for users to gain a sense of presence in the metaverse (Murray, 2020).

The results of this study confirm that the users’ imagination can significantly and positively influence their embodied presence in the metaverse, but not their embodied co-presence. In addition, the users’ imagination can positively influence their intention of continuous engagement through the serial mediation effects of embodied presence and co-presence. This suggests that imagination is currently an important factor that drive the use of the metaverse applications, and that imagination of a better future for the metaverse increases the users’ sense of embodied presence. Further, under the effect of embodied presence, the users establish a sense of embodied co-presence with others and eventually continue to engage in the metaverse, thus validating the ESP theory (Mennecke et al., 2011).

### Theoretical contributions

The results of this study have some theoretical contributions. First, this study provides the first empirical analysis to test the embodied presence theory in the context of metaverse applications. This study verifies that user psychological variables have a positive effect on Embodied Social Presence Theory but do not directly affect embodied co-presence. Additionally, this study confirms that imagination can be a psychological variable within the Embodied Social Presence Theory framework, which enriches the ESP theory.

Second, this study verifies that embodied co-presence can trigger the users’ intention to engage in the metaverse. Moreover, it finds that embodied co-presence cannot trigger the users’ intention to engage, which broadens the Embodied Social Presence Theory and enriches the metaverse research literature.

Thirdly, this study verifies that embodied presence can be used as a mediating variable for user psychology and continuous use intention, providing a favorable theoretical basis for explaining users’ usage behavior in virtual reality.

Finally, this study shows empirically that technical factors in embodied presence theory do not affect users’ intention to continuously participate in the metaverse, questioning the current development of the metaverse from the perspective of technical factors and enriching the research related to the metaverse field.

### Practical contributions

The results of this study have some practical implications. First, artificial intelligence techniques in metaverse applications should be improved. Artificial intelligence can improve the interaction ability of non-player characters (NPCs) in the metaverse. For example, NPCs in the metaverse can become autonomous through machine learning, thus enhancing the social authenticity of interactions in the metaverse and allowing users to gain a sense of embodied presence and co-existence by being more immersed in the metaverse.

Second, the quality of 3D modeling of the rendering technology can be improved. To make the metaverse a realistic immersive platform, the virtual environment needs to be constructed as consistent as possible with the real world. Companies that want to participate in metaverse development can build metaverse environments using 3D modeling tools, such as infrared depth scanning devices, to transpose real-world objects with fidelity into the metaverse. In addition, the development of metaverse environments needs to rely on the user generated content (UGC). Therefore, we should encourage the majority of users to participate in the 3D modeling of the metaverse. For example, individual users can use an iPhone LiDAR sensor to transfer elements from the real world into the metaverse in real time to enhance their sense of authenticity.

Third, the quality of the communication technologies can be improved. For the ubiquitous accessibility required by the metaverse, the users need highly synchronous and low-latency wireless communication technologies, which can allow users to have a perfect and smooth experience in real time and achieve a seamless link between the real and virtual worlds. It is recommended to enhance the rollout of 5G base stations and improve global 5G network coverage, thus promoting metaverse operational capability.

Finally, the development of any new industry to a certain stage may produce a certain bubble, such as the “economic bubble,” “real estate bubble,” and “the Internet bubble.” In social development, these bubbles can have drastic impacts due to unrealistic expectations; thus, we should help people understand the metaverse objectively, to prevent the adverse effects of excessive speculations.

### Limitations and future research

This study has the following limitations. First, the generalizability of the results is limited because this study was conducted with Chinese users and the sample size is not large enough to be representative. There are significant differences between Western and Chinese cultural contexts, etc., and such differences can affect users’ psychological and behavioral responses. Therefore, by testing our model in different cultural contexts using samples from other environments, other interesting findings may be observed.

Second, many applications claiming to be metaverse are more similar to “sandbox” games; thus, the results of this study need further validation. In particular, a full involvement with metaverse technologies by the users is not verified in this study. Therefore, further research is necessary with the gradual development of metaverse technologies.

## Data availability statement

The raw data supporting the conclusions of this article will be made available by the authors, without undue reservation.

## Author contributions

GZ and JC: conceptualization. GZ: methodology and writing—original draft preparation. JC: software and project administration. GZ and JQ: formal analysis. GZ, DL, and JC: writing—review and editing. All authors contributed to the article and approved the submitted version.

## Conflict of interest

The authors declare that the research was conducted in the absence of any commercial or financial relationships that could be construed as a potential conflict of interest.

## Publisher’s note

All claims expressed in this article are solely those of the authors and do not necessarily represent those of their affiliated organizations, or those of the publisher, the editors and the reviewers. Any product that may be evaluated in this article, or claim that may be made by its manufacturer, is not guaranteed or endorsed by the publisher.

## Footnotes

^1^ https://index.baidu.com

## Appendix

Appendix A

**Table:**

Factors	Serial Num.	Item	References
Interaction Quality (INQ)	INQ1	I think the quality of interaction with the environment in the metaverse is very good.	Joon Choi and Sik Kim (2013) and Kim and Ko (2019)
	INQ2	I feel that I have proper control over the content of the metaverse.	
	INQ3	I can freely and comfortably control my own perspective.	
	INQ4	I can experience the metaverse in an interactive way. (Drop)	
Rendering Quality (REQ)	REQ1	I think the sensory information provided in the metaverse is very vivid.	Kim and Ko (2019)
	REQ2	I think there is a wealth of sensory information available in the metaverse.	
	REQ3	I think the sensory content provided in the metaverse is very detailed.	
Communication Quality (COQ)	COQ1	The communication technology in the metaverse can effectively provide me with the feedback that I need.	Lowry et al. (2009)
	COQ2	The communication technology in the metaverse facilitates my communication with others.	
	COQ3	The communication technology in the metaverse provides me with the opportunity to communicate with others.	
Imagination (IMA)	IMA1	The information from the outside world makes me imagine that the metaverse is mysterious.	Choi et al. (2013)
	IMA2	The information from the outside world makes me think that the metaverse can satisfy my imagination.	
	IMA3	The information from the outside world makes me imagine that using the metaverse will give me special inspiration. (Drop)	
	IMA4	The information from the outside world makes me imagine that the metaverse will provide me with unprecedented experiences.	
Embodied presence (EPO)	EMP1	There is a sense of human interaction in the metaverse.	Bailenson et al. (2004) and Gao et al. (2017)
	EMP2	Socializing in the metaverse with a sense of reality.	
	EMP3	I can be aware of my presence in the metaverse. (Drop)	
	EMP4	In the metaverse, the incarnation is sentient and alive to me.	
Embodied co-presence (ECP)	ECP1	In the metaverse, I can feel the presence of other people.	Poeschl and Doering (2015)
	ECP2	In the metaverse, others can feel my presence.	
	ECP3	In the metaverse, I am not alone.	
Continuous engagement intention (COE)	COE1	I will regularly enter the metaverse.	Zhoc et al. (2018)
	COE2	I will regularly contact my friends in the metaverse.	
	COE2	I will regularly visit the metaverse to get information.	
